# Clinical predictors of hepatic complications in Anorexia Nervosa

**DOI:** 10.1192/j.eurpsy.2022.978

**Published:** 2022-09-01

**Authors:** S. Villa, I. Sánchez, F. Fernandez-Aranda, N. Custal, J. Menchón, P. Alonso

**Affiliations:** 1 Hospital de Bellvitge, Department Of Psychiatry, Barcelona, Spain; 2 Bellvitge University Hospital, Department Of Psychiatry, L’Hospitalet de Llobregat, Spain; 3 University Hospital of Bellvitge,-IDIBELL, Department Of Psychiatry, Barcelona, Spain; 4 Hospital Universitario de Bellvitge, Psiquiatría, Barcelona, Spain

**Keywords:** Anorexia, eating disorder, clinical predictors, hepatic complications

## Abstract

**Introduction:**

Hepatic ones are some of the most described somatic complications in anorexia nervosa (AN) affected patients. They can be due to malnutrition, which is the more usual thing, or due to re-feeding. The first one can lead to more marked elevations of the hepatic enzymes, especially alanine-aminotransferase (ALT). It’s been also described the relation between a sharply decreased body mass index (BMI) and this kind of complications, but there are still to determine more predictors.

**Objectives:**

Identifying clinical predictors of hepatic complications in AN.

**Methods:**

We analysed data from 71 AN affected patients hospitalized at Bellvitge Hospital from January 2016 to October 2021. We used IBM SPSS Statistics 22 to do all the statistics in this work.

**Results:**

The medium age of the sample was 27.66 years with 10.8 years of evolution of AN. The medium BMI was 13.88. 33.80% of them had some sort of hepatic enzymes elevation, two of them a several one. AST, ALT and ALP were significantly more elevated in those patients with lower BMI. GGT was significantly more elevated in patients with more years of disorder development. We didn’t identify correlation between any purgative method and hepatic alterations.

**Conclusions:**

The elevation of ALT, AST and ALP seems to be related with the BMI of the patients, while the elevation of the GGT turns out to be related to the time of evolution of the eating disorder. Purgative methods don’t seem to be related to the development of hepatic alterations in AN.

**Disclosure:**

No significant relationships.

